# Combining Virtual Reality and Machine Learning for Leadership Styles Recognition

**DOI:** 10.3389/fpsyg.2022.864266

**Published:** 2022-05-31

**Authors:** Elena Parra, Aitana García Delgado, Lucía Amalia Carrasco-Ribelles, Irene Alice Chicchi Giglioli, Javier Marín-Morales, Cristina Giglio, Mariano Alcañiz Raya

**Affiliations:** ^1^Institute for Research and Innovation in Bioengineering, Polytechnic University of Valencia, Valencia, Spain; ^2^Fundació Institut Universitari per a la Recerca a l’Atenció Primària de Salut Jordi Gol i Gurina, Cornellà de Llobregat, Spain

**Keywords:** leadership style recognition, virtual reality, eye-tracking, machine learning, leadership

## Abstract

The aim of this study was to evaluate the viability of a new selection procedure based on machine learning (ML) and virtual reality (VR). Specifically, decision-making behaviours and eye-gaze patterns were used to classify individuals based on their leadership styles while immersed in virtual environments that represented social workplace situations. The virtual environments were designed using an evidence-centred design approach. Interaction and gaze patterns were recorded in 83 subjects, who were classified as having either high or low leadership style, which was assessed using the Multifactor leadership questionnaire. A ML model that combined behaviour outputs and eye-gaze patterns was developed to predict subjects’ leadership styles (high vs low). The results indicated that the different styles could be differentiated by eye-gaze patterns and behaviours carried out during immersive VR. Eye-tracking measures contributed more significantly to this differentiation than behavioural metrics. Although the results should be taken with caution as the small sample does not allow generalization of the data, this study illustrates the potential for a future research roadmap that combines VR, implicit measures, and ML for personnel selection.

## Introduction

Leadership research includes a large number of theories and models that have evolved from personality models such as the great man theory (Carlyle, 1841; Galton, 1891; Bowden, 1926) or trait theory (Bingham, 1927), behavioural models that focus on the characteristics and behaviours of a leader, (Fleishman et al., 1955; Likert, 1961; Blake and Mouton, 1964) and broad models that acknowledge situational, contextual, communicational, and organisational factors such as the situational model (Hook, 1957; Stogdill, 1959) or the contingency model (Fiedler, 1964; Evans, 1970; Yukl, 1971). A more recent theory from Bass (1985), the transformational and transactional leadership theory, recognised the existence of three leadership styles: transactional, transformational, and passive-avoidant. Transactional leaders present behaviours highly focused on the achievement of objectives and characteristically engage in unilateral decision-making without involving team members. The transactional leader distributes tasks, establishes the guidelines to be followed, and monitors these. If the tasks are executed correctly, positive reinforcement is applied, however, punishments for errors and deviations are also applied, which have negative effects on subordinates (Limsila and Ogunlana, 2008). In contrast, transformational leadership attaches greater importance to relationships, motivation, and communication. Such leaders present positive images of themselves and others and express concern toward their employees and their personal and work-related problems. The ability of transformational leaders to express their opinions while respecting the rights of their subordinates also express their opinions is inferred. Transformational leaders will reinforce this behaviour by involving their teams in decision-making processes. Finally, passive-avoidant leadership is characterised by the total absence of leadership. According to previous studies (Valldeneu et al., 2021) leadership style is closely related to organizational results, innovation, success and recognition of the company. Specifically, it has been seen that leaders who tend to adopt a more transformational leadership approach and avoid passive-avoidance attributes could improve organizational outcomes and work engagement of employees (Valldeneu et al., 2021). Bearing in mind these data, it seems important to know before hiring the candidate his degree or level of connection with each of the leadership styles. In this way, companies could improve organizational outcomes by hiring leaders who are able to build trust in their followers, who inspire power and pride, and who become reference models for their followers, that is, leaders who present behaviours and attitudes typical of the transformational leadership style.

The Multifactor Leadership Questionnaire (MLQ; Bass and Avolio, 1996) is a well-validated self-report questionnaire used to measure these three styles of leadership and their influence on subordinates. It consists of 45 items: 36 items refer to leadership styles, and nine questions refer to three organisational outcome variables (extra-effort, leader effectiveness, and employee satisfaction; see Table 1).

**TABLE 1:** Factor structure of MLQ-5X.

Leadership style	Variables	Definition
Transformational leadership	(a) Idealised influence (Attributed) four items	Influence that the leader exerts on their followers promoting respect, trust, and admiration through the charisma that makes the leader perceived as safe and powerful
	(b) Idealised influence (Behaviour): four items	Actions carried out by the leader that focus on values, beliefs, and a sense of mission, which promote a high sense of self- identification with the leader (e.g., decision-making and considering moral and ethical aspects)
	(c) Inspirational motivation: four items	Leader’s ability to motivate their team members, provide meaning to their work and formulate an optimistic and attractive vision of the future (e.g., expressing confidence that the objectives will be achieved)
	(d) Intellectual stimulation: four items	Leader encourages team members to be innovative, creative, and seek the solution to problems for themselves. That is, they encourage personal autonomy, and value and trust their followers to solve problems (e.g., they ask for opinions of others)
	(e) Individualised consideration: four items	Willingness of the leader to know the aspirations, interests, and objectives of each of the subordinates, as well as promoting their achievement and individual growth (e.g., spending time getting to know people in the work team)
Transactional leadership	(f) Contingent reward: four items	Recognition and reinforcement from the leader for each employee when they meet the objectives.
	(g) Management-by-exception (Active): four items	Leaders who focus on correcting employee failures and deviations to ensure achievement of objectives.
Passive-avoidant leadership	(h) Management-by-exception (Passive): four items	Conservative leader who delays any decision-making that involves a change. The leader only intervenes when the seriousness of the problem is very evident.
	(i) Laissez-faire: four items	Total avoidance when dealing with important problems or decisions.
**Organisational results**		
	(a) Extra-effort: three items	The leader encourages greater participation from subordinates, who, in turn, are willing to work harder to achieve the objectives proposed by the group.
	(b) Effectiveness: three items	The leader is capable of optimising both material and human resources, achieving optimal results at low cost.
	(c) Satisfaction: three items	The actions of the leader generate gratification and cohesion in the group, which encourages the correct development of tasks.

Although self-reported questionnaires have traditionally been used for assessment in organisational leadership research, they present several limitations regarding ecological validity (Schmuckler, 2001), as they are decontextualised from real situations and do not elicit real-life behavioural responses. Furthermore, self-report measures are determined by human perceptions, and therefore social desirability and acquiescence biases may affect the veracity of responses (Nederhof, 1985; Furnham, 1986; Grimm, 2010). Additionally, there is a growing concern in contemporary academia regarding the effectiveness of such instruments and scales (Fisher and Chaffee, 2018; Crawford and Kelder, 2019). Some researchers have called for an analysis of existing leadership using other methodological evaluations to identify problems such as the halo effect, which fails to capture real behaviours (Baumeister et al., 2007), and threats to validity (Antonakis et al., 2010), which have also been a recent topic of interest (Crawford and Kelder, 2019).

In order to overcome these limitations, advances in immersive virtual reality (VR) technologies, combined with implicit measures such as behavioural decision-making, eye-gaze patterns, and machine learning (ML) techniques, are enabling the creation of experiences similar to real-life and are therefore able to better identify implicit behaviours and recognise leadership styles (Alcañiz Raya et al., 2018; Parra et al., 2021).

### Virtual Reality and Human Behaviour Assessment

Virtual reality can be viewed as a 3D synthetic environment able to simulate real-life experiences, where subjects can interact with their surroundings as if they were in the real world (Prat et al., 1995). The combination of various technological devices (visual, auditory, and haptic) and tracking systems that accurately reproduce stimuli creates a significant sense of presence. The user has the sensation of “being there,” and as a result, can forget that the situation is not real, and therefore behave (both cerebrally and physically) as if the VR experience were real life (Biocca, 1997; Slater, 2009; Pillai et al., 2013). These technologies allow information to be collected directly from the user in real-time (e.g., decision-making responses and times). Additionally, they also allow the integration and collection of other implicit measures, such as brain activity, skin conductance, cardiac variability, and eye-tracking. These measures provide valuable, indirect sources of information related to the implicit correlations of leadership competencies (Alcañiz Raya et al., 2018; Parra et al., 2021). This experience is complicated, or even impossible, to achieve in laboratory settings, as the use of multisensory laboratory stimulation does not present the complete, immersive, contextual experience that VR does. A recent review of social cognitive neuroscience and VR found that the use of this type of technology was effective with regard to affective induction, social psychology, and neuropsychological evaluation (Parsons, 2015). Furthermore, VR environments can increase user participation through “stealthy” assessment design approaches (Shute et al., 2016). The design and development of virtual environments requires a methodology that enables the stratification and determination of knowledge layers while incorporating valid measurements that enable the evaluation of evidence-based competencies. Advances in VR technology have enabled the capture of implicit measures without the need for subjects to self-report on aspects related to their capabilities. For stealth assessment methods, technologies based on evidence-centred design (ECD) have been used as valid and reliable reference frameworks for test design. ECD was developed primarily in the education field to improve the validity and reliability of test measures for students. ECD considers evaluations as evidence-based arguments. That is, actions from which one can observe what students say or do at a particular time and then infer what the students know, can do or have achieved (Mislevy et al., 2003). The ECD framework defines several interconnected models, three of which form the core of the framework and are relevant to the present study: competency, structure, and task.

• Competency model: this model describes the abilities or skills to be measured.
• Evidence model: the objective of this model is to determine which observations are optimal by providing evidence of what the designer wants to measure.
• Task model: the task model is responsible for defining the characteristics of the specific evaluation activities or tasks.

In the leadership research field, VR has been primarily used for training skills (Mainemelis and Ronson, 2006; Jones and Oswick, 2007; Rafaeli, 2010), as its efficacy in differentiating leadership styles has been limited (Parra et al., 2021). Moreover, leadership VR training has traditionally used non-immersive 2D graphical stimuli, characterised by flat graphics that limits the transferability of learned skills to the real world (Kato and de Klerk, 2017). It has been shown that immersive learning using immersive 3D virtual environments for training skills is more effective than 2D, due to the higher sense of presence that 3D VR offers (Dengel and Mägdefrau, 2019).

### Implicit Measures of Leadership Behaviour

There are many organisational behavioural theories that assume users have conscious control over their attitudes and actions (Ajzen, 1991). These approaches are based on traditional theoretical perspectives that consider humans capable of verbalising and being conscious of the brain processes involved in attitudes, emotions, and behaviours (Brief, 1998; Simon, 2013). In contrast to the traditional approach of using explicit measures, many neuroscience researchers (Barsade et al., 2009; Becker et al., 2011) have indicated that much of the processing related to behaviour, emotion, and attitude, within the context of work, occurs outside of consciousness, and therefore involves implicit processes that subjects themselves cannot verbalise due to their unawareness of them. Implicit processes can be defined as brain functions that occur automatically and outside of one’s conscious control or awareness, whereas explicit processes occur through conscious executive control (Becker et al., 2011). The link between explicit and implicit measures could lend greater veracity and validity when measuring behaviours in complex contexts, such as day-to-day work. Implicit measures can involve both brain and physiological measures, such as electroencephalogram (EEG; Balthazard et al., 2012), galvanic skin response (Nikula, 1991; Sequeira et al., 2009), heart rate variability (Massaro and Pecchia, 2019), and decision-making behaviours and eye-gaze patterns (Parra et al., 2021). By incorporating a balance of implicit and explicit measures in human resource management and organisational behaviour research, academics could develop more comprehensive and integrated theories of work phenomena. This study focuses on decision-making behaviours and eye-gaze patterns.

#### Decision-Making Behaviours and Eye-Gaze Patterns as Implicit Virtual Reality Measures

Rowe and Boulgarides (1983) decision style theory on leadership claims there is a relationship between a leader’s decision-making style and whether their leadership style is transactional or transformational. According to the theory of decision-making, an individual’s style of decision-making depends on how one understands and perceives a situation and how they respond to the contextual stimuli presented. Therefore, depending on their understanding of a situation, an individual may have a decision-making style that is focused on people-orientation and understanding the state of the team that surrounds them. However, an individual may also have a decision-making style that instead focuses on their decisions and on achieving objectives while the state of their team takes a back seat. Rowe and Boulgarides (1983) linked their typology of decision-making styles to individual needs for task or relationship orientation, a posture more suited to the standards of a transformational leader. Further, it has been postulated that managerial decision-makers are primarily driven by their need for power, while behavioural decision-makers are concerned about the need for affiliation (Martinsons and Davison, 2007; Torres and Augusto, 2017). Because those who make managerial decisions typically have little tolerance for ambiguity, they incidentally have a strong desire for structure, rules, and procedures (Leonard et al., 1999). This is considered similar to the behaviour that a leader who is focused on achieving objectives would exhibit, focused more on a transactional style of decision-making and not taking into account the state of their team (Halpin and Winer, 1957). This tendency leads such leaders to be inclined to make directive decisions, such as giving clear orders to subordinates and executing decisions themselves. Conversely, behavioural decision-makers are concerned with maintaining good relationships through offering psychological support and encouragement to their teams during complex situations, making collaboration and direct relationships with the team the basis of their leadership style, and thus, corresponds directly with the transformational style (Leonard et al., 1999). An indication of this style of decision-making is that it involves consistently communicating with teams and seeking and using their comments in the final decision-making.

Social gazing patterns refer to the implicit and automatic tendency of people to focus their attention on the behaviours of others and interpret the relevant social signals (Meibner and Oll, 2019). According to social attention theory, visual attention allows people to recognise each other, communicate their mental states, and predict the behaviour of others (Frischen et al., 2007). This has potential relevance when it comes to solving problems in group situations, such as selecting a leader. Evidence shows that people can predict leadership cues by watching silent voice clips (Tskhay et al., 2017; Gerpott et al., 2018). Similarly, people can perceive differences in visual patterns during presentations. The visual behaviour of audiences has been shown to be modified based on the charisma of the leader presenting (Maran et al., 2019). Specifically, a relationship has been found between influential leadership and direct gaze (Maran et al., 2019), from which the existence of different gaze patterns between the three leadership styles is inferred. Eye-tracking techniques provide two different indicators, the orientation of attention toward someone or something through the number of fixations and the maintenance of this attention throughout the duration of fixations. This means these techniques can monitor where attention is focused initially and automatically, to which stimuli, and in how this visual attention is maintained. Therefore, this type of measurement allows the analysis of the in-depth internal processes of an individual’s visual attention in social situations and complex simulations.

The ECD model for VR and implicit measures are promising tools and methods for the assessment of leadership styles, as they enable the collection of large amounts of real-time data relating to things such as eye-gazing, task execution, decision-making behaviours, and latency times. The analysis of this data can be complicated due to the amount and heterogeneity of the data. ML has emerged as an effective method to analyse large amounts of data. In the current study, ML methods were used to obtain predictive data regarding leadership styles.

### Machine Learning and Organisational Behaviour

Machine learning is a scientific discipline within the artificial intelligence (AI) field that deals with the design and development of algorithms that allow computers to evolve behaviours based on empirical data, recognise hidden patterns, and use them to make predictions (Mikalef et al., 2018). Recently, an increasing number of researchers have noted how ML techniques applied to big data can be used to study individuals behaviours in workplaces (George et al., 2014). Some leading companies have started to use AI techniques, such as ML, to automate decision-making processes, improving the processes by increasing employee involvement and customers satisfaction (Wellers et al., 2017). Other recent studies are proposing the use of ML as predictive models in organisational environments. For example, Na and Kim (2019) used a ML algorithm to predict the impact of disease on returning to work. Other studies have used ML to predict employee performance (Kirimi and Moturi, 2016), employee turnover (Oliveira et al., 2019), and evaluate job candidates (Faliagka et al., 2012). Furthermore, in leadership studies, ML has been used to identify traits that define the leadership role (Doornenbal et al., 2021) and measure personality traits (Hrazdil et al., 2020). The use of ML techniques for implicit measures gathered within virtual environments has occurred primarily in clinical psychology (Alcañiz Raya et al., 2020a,b) and less in organisational situations (De-Juan-Ripoll et al., 2021).

The aim of this study was to recognise transformational, transactional, and passive-avoidance leadership styles while exposed to a 3D virtual environment that simulated workplace social interactions. Decision-making behaviour and eye-gaze tracking were used as implicit measures. Additionally, ML methods were used in the analysis of the implicit measures to explore if it is possible to discriminate between transformational, transactional, and passive-avoidance leadership styles and to identify the parameters that best discriminate between these styles. The main hypotheses were that participants’ decision-making behaviours during the VR experience would be able to indicate their leadership styles and that participants’ eye-gaze patterns during the VR experience would also indicate leadership style.

## Materials and Methods

### Participants

The study sample consisted of 83 subjects, of which 32 were women and 51 were men (*M* = 42, SD = 3.44). The selection of the same was subject to a selection carried out through work criteria, in which they had to have a team under their charge for at least 1 year, considering that leading a team entails certain leadership skills. In the same way, a set of students was included, which had non-leadership criteria, based on the fact that they had not previously had teams under their charge. The fact of including a group of students in the sample, in addition to providing the lack of experience in leading teams, balanced the possibility of finding a very high level of specific leadership styles. In the same way, the MLQ was administered to them to determine the leadership style in the same way as in the rest of the sample, since in the absence of experience, leadership would appear as an inherent trait in the user’s behaviour. The sample was completely Caucasian, all of Spanish nationality and Spanish speaking. The inclusion criteria for participation in the studies were that they were of legal age, had a team under their care for at least 1 year if in the sample of professionals, or had not had a team under their care, or prior work experience, if in the student sample. Individuals were excluded if they had any type of mental disorder or took medication that affected their cognitive and mental functions. This sample included leaders or professionals from a wide variety of industries, including pharmaceutical, banking, and consulting.

The sample was counterbalanced at the leadership level through the results obtained in the MLQ questionnaire. Thus, a complete representation of each of the three leadership styles was obtained based on the responses to the questionnaire.

The sample was counterbalanced in terms of gender and familiarity with the use of video games. Additionally, the level of leadership or human resource management an individual carried out in their workplace was considered.

All participants submitted their written consent to participate in the study. The study was carried out in accordance with the Declaration of Helsinki (1964), and was approved by the ethics committee of the Polytechnic University of Valencia, Spain.

### Leadership Assessment

The MLQ-Leadership form was used for each leader, while the MLQ-Subordinate form was used for a subordinate of each leader. This has become one of the most widely used instruments to measure leadership in the field of organisational psychology (Moreno-Casado et al., 2021). The questionnaire describes the leadership style that the person perceives themselves to have or that the subordinate thinks the leader has. It consists of 45 items that were rated on a five-point Likert scale. For each leadership style, there are different dimensions measured. Transformational leadership has five dimensions: idealised influence (attributed), idealised influence (behaviour), inspirational motivation, intellectual stimulation, and individualised consideration. Transactional leadership has two dimensions: contingent reward and management by exception (active). Passive-avoidant leadership also has two dimensions: management by exception (passive) and Laissez-faire. In addition, the questionnaire analyses the effect of leadership on organisational outcomes across three factors: extra-effort, effectiveness, and satisfaction. Specifically, nine questions are related to these three organisational outcome variables, while 36 questions are related to the leadership styles, consisting of questions specific to each of the nine aforementioned dimensions that exist within the different leadership styles.

### Virtual Environment Modelling

**FIGURE 1:**
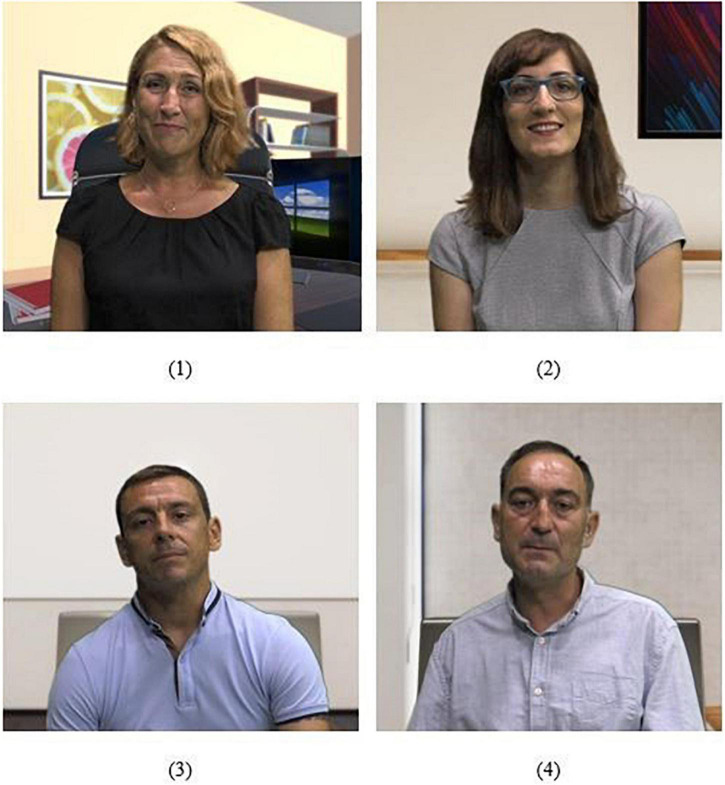
Virtual agents’ characterisation (1) transactional leader, (2) transformational leader, (3) transactional leader, and (4) passive-avoidant leader.

To create a valid measure to obtain reliable results from the VR experience, ECD guidelines were followed. Following these guidelines, a story narrative was designed, with different scenes set in different office environments. Specifically, it consisted of an office meeting room environment, where a series of dynamics are developed with other avatars, in which the participant must make decisions and carry out behaviours that determine the subsequent development of the scenes. The VR involved four adult virtual agents (two women and two men) that were designed with personality traits and competencies according to the transformational, transactional and passive-avoidant leadership styles (Figure 1). Specifically, one of the characters was defined as an organiser, another as emotional-interpersonal, another as logical, and the last as non-interventional:

(1) The organiser virtual agent (woman) is characterised by planned, sequential and structured thinking (transactional leadership). Her role focuses on exposing the issues, and she is the one who decides what to do but does not get involved either positively or negatively.
(2) The emotional-interpersonal or communicative virtual agent (woman) is characterised by presenting empathic traits, interpersonal warmth, fluid communication, and holistic thinking (transformational leadership). This agent talks about the topic to be discussed with confidence in herself and in the team and encourages everyone to be part of reaching a consensual decision. This character is approachable and respects the opinions of others.
(3) The logical virtual agent (man) is characterised by presenting mathematical, technical, and analytical reasoning, with a tendency toward negativity (transactional leadership). This agent sets clear standards to follow and dishes out punishment for any mistakes made.
(4) The last of the agents (man) is characterised by non-intervention. He avoids giving any kind of feedback regarding his opinion, leaves the decision in the hands of the team, and can be highly upset depending on the situation (passive-avoidant leadership).

The VR experience consisted of four different situations, in the appendices we have put a functional diagram of each situation. At the beginning of each situation, a problem was presented by one of the virtual agents to the other agents and the participant. In each situation, there were two to three problems on the agenda to be resolved among those attending the meeting. To find a solution to each of the problems, the participant had to make various decisions freely by voice and by selecting the option that aligned with their opinion. Each decision made led the story narrative down a different path. Specifically, mini-games were designed for some decisions, but not every decision leads to a mini-game. The option to access the games depended on the decision style, being more proactive the cases where the user finally accessed the games. Each possible decision was developed according to a systematic method based on three decision-making behaviours, communication (a), control (b), and avoidance (c):

(a) Communication refers to decision-making in which all team members are involved, where information regarded the opinions of the other team members is sought out and collected. It implies a desire to be open and accessible and to collect information of both a professional and personal nature. This style of decision-making is based on approach behaviours; new situations are seen as challenges and are faced with optimism. It is a decision-making style that is related to transformational leadership.
(b) Control is a type of decision-making where an individual takes initiative without asking the opinion of the other team members and subsequently distributes and controls tasks and their development. It is a behavioural style associated with transactional leadership.
(c) There is the possibility that the participant opts for the option of avoiding by doing nothing to solve the problems raised. The behaviour of this participant is related to a passive-avoidant leadership style. This type of leadership is characterised by delaying or avoiding decision-making and by delegating responsibilities to other team members.

**FIGURE 2:**
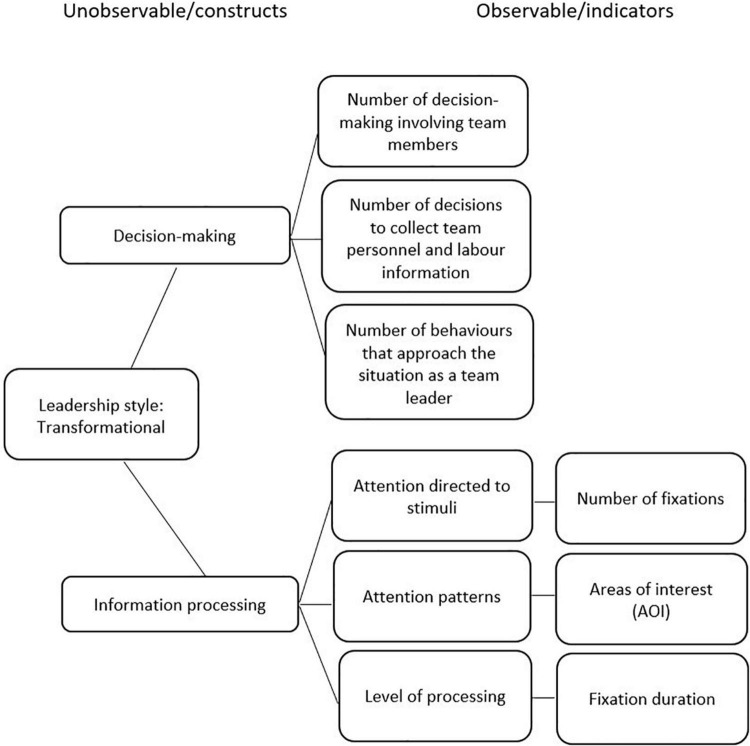
Transformational leadership.

**FIGURE 3:**
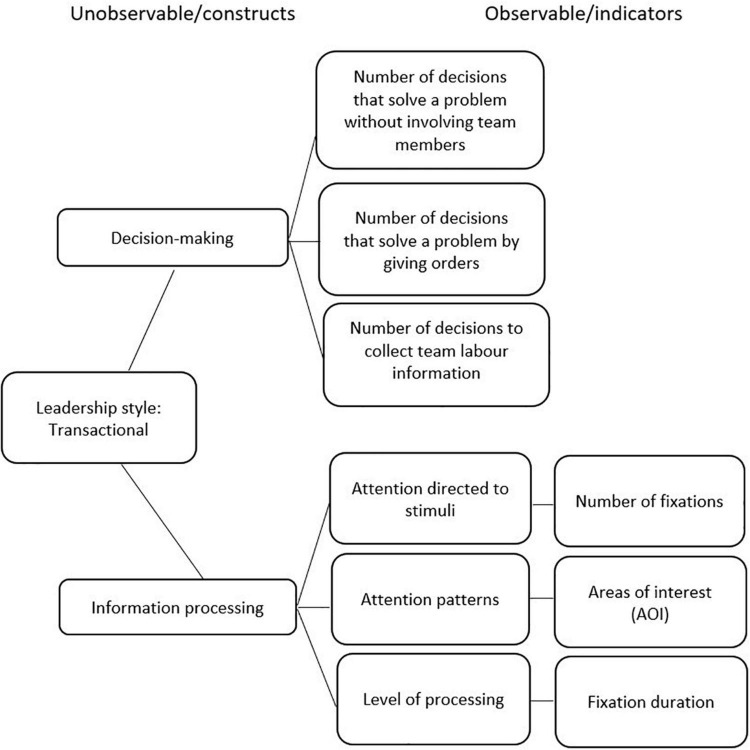
Transactional leadership.

**FIGURE 4:**
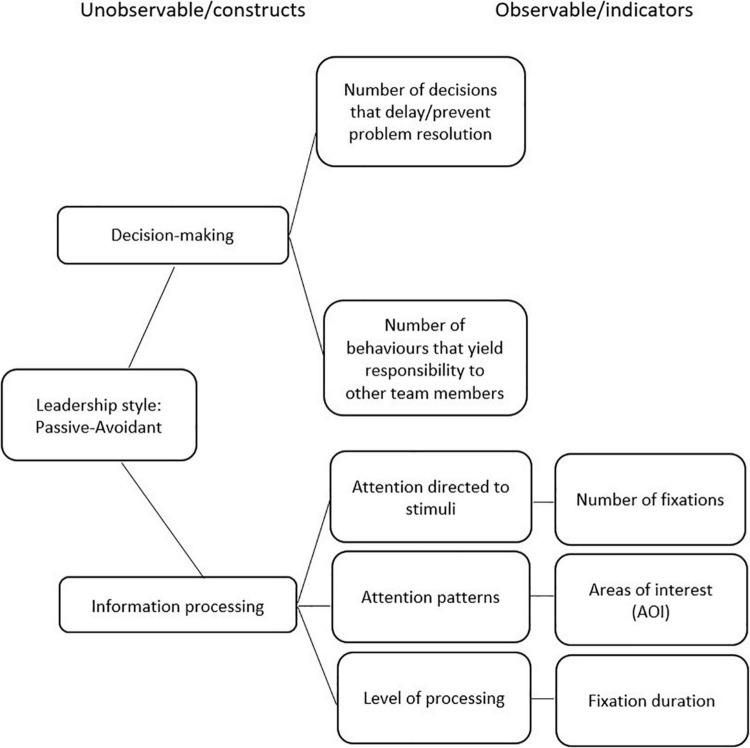
Passive-avoidant leadership.

The four situations that were developed in VR were designed in accordance with the theoretical framework of reference (transformational, transactional and passive-avoidant leadership styles), the ECD model, and the MLQ instrument. Figures 2–4 display the competency models according to each leadership style and their relative indicators. For each style, a graphical model of the indicators (observable tasks, data collected from user performance, and unobservable, theoretical leadership constructs) is presented.

### Experimental Procedure

To determine the leadership styles present within the sample, the participants completed the MLQ online. A short demographic questionnaire was also completed by each participant to collect data related to age, gender, and job position. Following the completion of these, participants visited the laboratory to complete the experimental phase of the study. The experimental phase consisted of a single 1.5-h session in which the participants experienced a simulation in an immersive VR environment. The first 2 min of the experience showed a brief tutorial explaining how to use the virtual environment. The room in which the experimentation took place was a neutral experimental room so that distractions could be avoided. At the beginning of the session, the eye-tracking application was started manually, and calibration was carried out once the head-mounted display (HMD) was placed on the participant. After these steps, the virtual environment simulation began and the first 2 min of the experience showed a brief tutorial explaining how to use the virtual environment. After that, the user is immersed in the first scene of the first situation, that is, the office. Once the first situation is overcome, the next situation begins, until completing the entire experience (four situations in total). The average execution time of the experience was 1.5 h. The longest execution time was approximately 1 h and 48 min, and the shortest was approximately 1 h and 10 min.

The visual attention was measured using the HTC Vive Pro Eye HMD, with a combined resolution of 2,880 × 1,600 pixels (1,440 × 1,600 per eye), a 110° field of view, and a refresh rate of 90 Hz. The application of VR was carried out on the MSI GE75 Raider 9SF-1204XES, 17.3” laptop (i7-9750H, RAM 32 GB, 1 TB NVMe PCIe Gen3x4 SSD, GeForce RTX 2070 GDDR6 8GB).

The VR system was developed using Unity 5.5 1f1 software, applying C# pro254 programming language with the Visual Studio tool.

### Data Processing

Three different data sources were available: behavioural data (i.e., decisions made by the participant in the VR experience), eye-tracking data (i.e., sight fixations during the VR experience), and questionnaire answers that collected the psychological variables to study (e.g., MLQ scores). From the behavioural data and eye-tracking data, several variables were processed, Table 2 presents a detailed description of the behavioural variables. A total of 63 variables were obtained from behavioural data. If a participant did not complete all VR game types or had missing values in the variables that measured performance and motive during the games, and there was more than one possible option to choose from, they were marked as “not chosen.” A total of 110 variables were extracted from eye-tracking data, as shown in Table 3.

**TABLE 2:** Description of the variables obtained from the decisions made during the VR experience, including the related psychological trait of each one.

Number of variables	Description
5	Location the participant chooses on the meeting table (1 × Situation + Mode)
16	Use of the messaging app: utility rates given (13), number of times open, number of messages sent, and real interaction (messages open minus messages sent)
18	Per type of mini-game (6×): number of times chosen, self-rating of the performance, and reported reason for choosing the mini-game

**TABLE 3:** Description of the variables obtained from the eye-tracking data.

Type of variable	Number of variables	Description
Fixations	10	Mean number (and standard deviation) of fixations done per situation, and total during the whole VR experience.
Sx_Participant_VirtualAgent	20	Per situation (4×), the average number of times the participant looks at each virtual agent (6×) while the participant is speaking.
Participant_VirtualAgent	5	Over the entire experience, the average time spent by the participant talking, looking at each virtual agent (6×).
Sx_VirtualAgentA_VirtualAgentB	60	Per situation (4×), the average number of times the participant looks at a virtual agent B while virtual agent A is speaking (6×).
VirtualAgentA_VirtualAgentB	15	Over the entire experience, the average number of times the participant looks at a virtual agent B while virtual agent A is speaking.

*The environment is considered as another possible area to gaze at while speaking, so the time spent looking at the environment is also calculated as if it were a virtual agent itself.*

### Statistical Analysis

Three participants did not complete the MLQ-Leadership form, while 12 did not complete the MLQ-Subordinate form. As a result, their data were excluded from the analysis. A multivariate outlier analysis (Filzmoser and Viertl, 2004) was performed to detect and remove any participant whose scores in each questionnaire could be considered extreme. In order to detect these participants, the Mahalanobis distance between participants was calculated using the numeric score on each of the seven subscales of each questionnaire. The probability of this distance belonging to a Chi-square distribution was calculated. If this probability was below 0.01, the participant’s scores were defined as outliers, and the participant was excluded from further analysis. One participant who completed the MLQ-Leadership form was considered an outlier, while two participants who completed the MLQ-Subordinate form were considered outliers. These participants were excluded only for the analysis of the variable in which their results were considered extreme. 77 participants’ MLQ-Leadership scores and 68 participants’ MLQ-Subordinate scores were analysed.

The description of the target data (i.e., MLQ scales) was performed by obtaining the mean, median, minimum, maximum, standard deviation, and interquartile range for each scale. The normality of the scores was studied through a Shapiro–Wilk test. All 14 MLQ subscales were categorised into high or low scores depending on the median of each variable, as most of them were non-normally distributed (*p* < 0.05, Shapiro–Wilk test). This was a necessary step for building the ML models described in the following section.

### Machine Learning

Multifactor leadership questionnaire recognition models were built using ML models and the data recorded during the VR experience. First, a feature selection was performed to reduce the dimensionality without defining any maximum limit of features. This feature selection was performed using a backward sequential wrapper (Doak, 1992). This method builds a model with all the available features using the selected ML algorithm and measures its performance. Then, at each subsequent step, a feature is removed, the model trained, and its performance measured. The feature where removal increased the performance measure most significantly (i.e., Cohen’s kappa) was removed from the set of features that will be used in the next step. After several repeat steps where the performance metric does not vary by more than 0.01, the process stops.

Multiple ML algorithms were used to obtain the best set of features, namely Random Forest, SVM, Naïve Bayes, XGBoost, and kNN. No further hyperparameter tuning was done. The default hyperparameters defined in mlr package v2.14.0 were used. After obtaining the best set of features for each ML algorithm, the model was trained and validated, and its metrics were calculated (i.e., accuracy, Cohen’s kappa, sensitivity true positive rate and specificity true negative rate). Cohen’s kappa is used since it considers chance agreement as a baseline in a single metric, in contrast to accuracy, which can be highly affected in unbalanced models. A threshold of 0.4 is considered as a threshold to consider a model as “good” (Kirch, 2008). Each step used repeated cross-validation (fivefold, four times), so the validation metrics correspond to the mean value across the 20 repetitions. Data from 10 participants were excluded from this model building process and only used as a test set. The test set was randomly chosen from all the participants who had scores from both MLQ scales available and was used to test the models for all the subscales. Both the statistical and ML analyses were performed in R (version 3.6.1).

## Results

### Multifactor Leadership Questionnaire Scores Description

Table 4 shows the description of scores on the different MLQ-Leadership and MLQ-Subordinate subscales. The distribution of all scores, except the MLQ-Leadership Transactional subscale scores, were non-Gaussian. Therefore, the median value was used to divide the scores into high or low scores. The balance between both categories varied from 50% in the MLQ-Subordinate Transformational subscale to 77% in the MLQ-Leadership Satisfaction subscale (i.e., 59 high-scoring participants vs 18 low-scoring participants).

**TABLE 4:** Description of the scores obtained by the participants in each of the MLQ-Leadership and MLQ-Subordinate subscales.

Variable	*N*	Mean	Median	Standard deviation	Interquartile range	Minimum	Maximum	Shapiro–Wild normality test *p*-value	High score (*N*)	Low score (*N*)
MLQ-Leadership transformational	77	12.08	12.2	1.74	2	5.8	15.4	0.002	41	36
MLQ-Leadership transactional	77	10.4	10.5	2.02	2.5	5	14	0.061	43	34
MLQ-Leadership passive-avoidant	77	3.42	3	2.44	2	0	12	<0.001	47	30
MLQ-Leadership laissez	77	1.7	1	1.72	3	0	7	<0.001	51	26
MLQ-Leadership effort	77	8.64	9	1.81	3	4	12	0.001	50	27
MLQ-Leadership effectiveness	77	11.94	12	1.73	2	8	16	0.006	51	26
MLQ-Leadership satisfaction	77	5.96	6	0.99	0	3	8	<0.001	59	18
MLQ-Subordinate transformational	68	10.52	11.7	3.24	4.1	0	15.4	<0.001	34	34
MLQ-Subordinate transactional	68	9.38	10	3.19	3.62	0	14.5	0.012	35	33
MLQ-Subordinate passive-avoidant	68	4.99	5	3.17	5	0	13	0.022	36	32
MLQ-Subordinate laissez	68	4.12	3	4.05	7	0	16	<0.001	39	29
MLQ-Subordinate effort	68	7	7	3.34	4	0	12	0.004	39	29
MLQ-Subordinate effectiveness	68	10.74	12	3.91	5.25	0	16	0.001	36	32
MLQ-Subordinate satisfaction	68	5.37	6	2.12	3	0	8	0.001	36	32

*The last two columns show the number of participants in each category after discretizing the scores according to the median value.*

### Multifactor Leadership Questionnaire Recognition Models

Table 5 shows the metrics and characteristics of the best model achieved for each MLQ subscale following the validation process. For every subscale, except for the MLQ-Subordinate Effectiveness subscale, models with validation accuracy above 0.7 and kappa above 0.4 were achieved. These results were mostly maintained within the test set, with all but five models maintaining validation accuracy above 0.7 and kappa above 0.4. The best-modelled subscales in terms of validation and test results were the MLQ-Leadership Transactional, MLQ-Subordinate Transformational, MLQ-Subordinate Passive-Avoidant, and MLQ-Subordinate Satisfaction subscales, as they each achieved kappa values above 0.5 in both sets. The number of selected variables for the models varied between 14 and 43, with most of the variables coming from the eye-tracking data set (i.e., the percentage of behavioural data included in the models varied from 0 to 32%).

**TABLE 5:** Metrics of the best ML model achieved for each MLQ subscale, both for the validation and the test set.

Subscale	Model	Features (*n*)			Validation set					Test set				
		Eye-tracking	Behavioural	Total	Accuracy	Kappa	AUC	TPR	TNR	Accuracy	Kappa	AUC	TPR	TNR
MLQ-Leadership transformational	kNN	20	3	23	0.78	0.53	0.74	0.8	0.76	0.69	0.4	0.67	0.57	0.83
MLQ-Leadership transactional	Naïve Bayes	13	7	20	0.84	0.66	0.88	0.8	0.9	0.75	0.53	0.83	0.57	1
MLQ-Leadership passive-avoidant	kNN	21	9	30	0.81	0.63	0.86	0.79	0.87	0.67	0.31	0.74	0.71	0.6
MLQ-Leadership laissez	RandomForest	14	0	14	0.74	0.4	0.82	0.88	0.53	0.75	0.31	0.59	1	0.25
MLQ-Leadership effort	kNN	33	5	38	0.84	0.65	0.84	0.8	0.89	0.75	0.47	0.8	0.75	0.75
MLQ-Leadership effectiveness	Naïve Bayes	19	4	23	0.81	0.61	0.8	0.81	0.85	0.67	0.4	0.91	0.5	1
MLQ-Leadership satisfaction	kNN	21	7	28	0.87	0.42	0.82	0.97	0.48	0.92	0.63	0.62	1	0.5
MLQ-Subordinate transformational	kNN	15	4	19	0.78	0.55	0.82	0.76	0.84	0.91	0.82	1	1	0.83
MLQ-Subordinate transactional	kNN	21	2	23	0.82	0.62	0.88	0.79	0.89	0.5	0	0.5	0.5	0.5
MLQ-Subordinate passive-avoidant	kNN	12	7	19	0.81	0.61	0.88	0.79	0.86	0.82	0.65	0.93	0.67	1
MLQ-Subordinate laissez	RandomForest	29	14	43	0.72	0.43	0.82	0.79	0.67	0.67	0.27	0.46	0.86	0.4
MLQ-Subordinate effort	Naïve Bayes	10	5	15	0.86	0.7	0.88	0.87	0.86	0.5	0.08	0.66	0.29	0.8
MLQ-Subordinate effectiveness	kNN	16	2	18	0.68	0.34	0.71	0.74	0.64	0.67	0.33	0.72	0.5	0.83
MLQ-Subordinate satisfaction	kNN	26	12	38	0.8	0.57	0.81	0.81	0.76	0.91	0.81	0.97	1	0.8

*The number of variables used by each model is divided according to their source (i.e., eye-tracking, or behavioural data). The values shown per metric in the validation set are the mean values of the cross-validation iterations. TPR and TNR stand for true positive rate and true negative rate, respectively.*

## Discussion

This study based on a multi-method approach, aims to offer a first approximation for the discrimination of different leadership styles through the joint use of VR and implicit measures, based on the results obtained in the MLQ questionnaire. Specifically, an immersive VR environment based on ECD was used in conjunction with eye-tracking measures. The use of these tools enabled behavioural decision-making in the virtual environment to be recorded, as well as the compartmental signals associated with eye-tracking for each of the three leadership styles. ML was used to build different models based on the two sources of information recorded during the VR environment experience. The main objective of this study was to replicate the MLQ classification based on implicit measures and in a VR environment. The simultaneous use of implicit measurements and VR allows an objective evaluation of leadership behaviour.

This methodology improves the ecological validity compared to the self-report measures since it enables behaviours and decision-making to be captured in scenarios that simulate real management situations.

The study includes an analysis of the frequency distribution (high vs low) of the different leadership styles to investigate the differences between the leadership styles, using a wide set of ML models that were based on decision-making behaviours and gaze tracking patterns.

### High and Low Transformational, Transactional, Passive-Avoidant, and Laissez-Faire Differences Between Measures

The first objective was to identify the differences between the different leadership styles both in the traditional measure questionnaire and in the VR experience. The results of the traditional measure indicated that, for the leader’s self-report form, 53% of participants had a high score in the transformational subscale, 56% in transactional, 61% in passive-avoidant, and 66% in Laissez-faire. Regarding the questionnaire completed by subordinates, 50% of participants had a high score in transformational leadership, 51% in transactional leadership, 53% in passive-avoidant, and 57% in Laissez-faire. Regarding the organisational results of the self-report, 71% of the sample were classified in the “high” category for the Extra-effort variable, 73% for Effectiveness, and 84% for Satisfaction. Of the results obtained from the subordinate questionnaire, 57% of the sample were classified as “high” for Extra-effort, 53% for Effectiveness, and 53% for Satisfaction.

These results indicate that the traditional evaluation measure can define and classify leadership styles. Furthermore, they indicated that there were a greater number of participants with high scores than low scores in all types of leadership and all the variables of the organisational results.

Regarding the VR experience, the results indicated that the different styles could be differentiated by the eye-gaze patterns and behaviours carried out during immersive VR. However, according to the results, ML models selected more variables from eye-tracking than from behavioural data, as eye-tracking was represented between 64 and 100% in the selected variables for all models. This indicates that there was a more significant contribution of information from the eye-tracking metrics. As such, the results indicate that the eye-tracking pattern is a more relevant and distinctive aspect of the different leadership styles compared to decision-making or behaviours carried out in VR immersion. This could explain why previous studies have focused on the gaze pattern to identify the peculiarities among leaders and their impact on organisations (Shim et al., 2020), as the gaze reflects complex mental states such as intentions, thoughts, beliefs, emotions, desires, and characteristics of social interaction (Frischen et al., 2007).

Looking at the ML models metrics, it is inferred that the capacity of the virtual environment to provoke behaviours (eye-gaze patterns and behavioural decision-making) enables the classification of participants according to their leadership style.

It should be noted that this study achieved homogeneous results between validation and test, with the exception of the results in five models. The leadership recognition models for the different leadership styles and organisational outcomes achieved accuracies between 78 and 87%. In addition, ML models were balanced in terms of sensibility and specificity in all cases except for Leadership-Laissez and Leadership-Satisfaction, suggesting each group of styles was able to be precisely identified. In terms of test set, this balance is not achieved, but this can be due to it includes 10 samples. Further analysis need to be done increasing the sample size of the test set. Moreover, the backward sequential wrapper implemented allows to easily explore the importance of each feature, in contrast to other dimensionality reduction techniques such as principal component analysis.

### Theoretical Implications

The use of a VR environment together with a non-intrusive method (eye-tracking) and ML for the evaluation of behavioural responses in complex situations can increase the knowledge about the attentional behavioural patterns and decision-making processes carried out by leaders with different leadership styles. Unlike most evaluations that use subjective self-report measures, this method combines neuroscience with VR, which, in turn, attributes greater objectivity and ecological validity to the results. With regard to implicit measures, previous studies have tried to identify leaders based on gaze-following behaviour among group members (Gerpott et al., 2018). In addition, ML models as a leadership classification tool have previously been used to carry out predictive analysis of eye-tracking behaviour during social interactions in non-immersive environments (Capozzi et al., 2019). However, unlike previous studies, the present study did not require the participation of other team members for leadership evaluation. Only the leader’s participation in the VR experience was required. This enabled the necessary data to be collected and analysed by ML to identify the participants according to their leadership styles. In addition, to evoke the typical behaviours associated with the leadership style of the participants, office spaces were recreated with high-pictorial realism and used hyper-realistic avatars. All these factors constitute an important contribution to Gerpott et al. (2018), which focused on the gaze pattern of leadership during social interactions to differentiate between leaders and non-leaders. Additionally, this study puts into practice the taxonomy proposed by Meibner and Oll (2019) and used the suggested ET measures, which are the number and duration of fixations required to capture the psychological and behavioural characteristics of the different leadership styles, as proposed by Parra et al. (2021). From this, the importance of non-verbal cues in the identification of leadership characteristics in organisations is inferred, as they cannot be evaluated through explicit measures.

### Practical Implications

Expanding the knowledge about the neuropsychological aspects responsible for the behaviours of individuals can constitute the basis for the modification and training of effective leadership behaviours *via* interventions promoting them. Such changes will be motivated by training and consolidated due to neuroplasticity, enabling the learning of new ways of behaving and making decisions (Sivalingam et al., 2017). Implicit measures can play an important role in the evaluation of behaviour and psychological leadership constructs (Dixon and Webb, 2017). As such, they can be used to evaluate the results of the behaviour training within the context of effective leadership and employee satisfaction. Examples of the implicit measures by which these changes can be evaluated are fMRI, qEEG, and eye-tracking. If these measures are to be used together or with VR systems, the use of ML algorithms that enable the analysis of large data sets may be beneficial, as they can facilitate the evaluation and interpretation of the results obtained, thereby promoting the advancement of the neuroscientific study of leadership.

## Limitations and Future Directions

In this study, a small sample size (<70) was observed and therefore, the size of the test set for the ML models was also small. This could have affected the results due to variability and, therefore, compromised the generalisability of the theory. However, the objective was not to design a new tool to identify leadership at a general level but to ascertain if, through the use of VR and eye-tracking, it was possible to replicate the classification of leadership styles of an explicit measure within a specific population. This goal has been achieved through the use of ML, which provided a predictive classification model. Regarding future directions, this work can serve as the basis for the study of leadership using novel technology, such as VR and ML, and implicit measures. Furthermore, this methodology can be applied for the evaluation of other important aspects of leadership at the cognitive, behavioural, psychological, and social levels.

## Conclusion

In this study, VR, implicit measures, and technological methods were used to evaluate three different leadership styles, transformational, transactional, and passive-avoidant. The combination of these methods, consisting of an immersive VR system, eye-tracking, and ML, offers a novel perspective on the study of leadership and the ability to replicate the results of the MLQ. Specifically, a VR environment was used to record the behaviour of each participant. Subsequently, ML was used for the analysis of the large dataset gained from the measurement of eye-tracking and decision-making during the VR experience. From this dataset, it was possible to develop different models capable of categorising each participant according to their leadership style. Therefore, the main contribution of this study is that it offers a multi-method approach that enables the capture and analysis of behavioural leadership variables and is able to classify these variables into different leadership styles.

## Data Availability Statement

The raw data supporting the conclusions of this article will be made available by the authors, without undue reservation.

## Ethics Statement

The study was conducted according to the guidelines of the Declaration of Helsinki and approved by the Ethics Committee of the Polytechnic University of Valencia (Protocol code: P01_08_07_20). The patients/participants provided their written informed consent to participate in this study. Written informed consent was obtained from the individual(s) for the publication of any identifiable images or data included in this article.

## Author Contributions

EP and MA: conceptualization, methodology, and resources. EP: validation and investigation. LC-R and JM-M: formal analysis and data curation. AG, CG, EP, IC, and LC-R: writing—original draft preparation. AG, IC, LC-R, EP, and JM-M: writing—review and editing. IC and MA: supervision. All authors contributed to the article and approved the submitted version.

## Conflict of Interest

The authors declare that the research was conducted in the absence of any commercial or financial relationships that could be construed as a potential conflict of interest.

## Publisher’s Note

All claims expressed in this article are solely those of the authors and do not necessarily represent those of their affiliated organizations, or those of the publisher, the editors and the reviewers. Any product that may be evaluated in this article, or claim that may be made by its manufacturer, is not guaranteed or endorsed by the publisher.
