# Supplementary material for: Combining Virtual Reality and Machine Learning for Leadership Styles Recognition
Source: Front Psychol. 2022 May 31;13:864266. doi: 10.3389/fpsyg.2022.864266 (PMC9197484; doi:10.3389/fpsyg.2022.864266)
Supplement: Supplementary file 1 [file Data_Sheet_1.PDF]

All situations have the same structure, taking into account that each situation presents differences with respect to its content: different meeting points, message topics and types of games. The emotional charge also differs in the situations: Two of the four situations present neutral emotional content (1 and 4) and two of them present high emotional charge (2 and 3). The following videos show the development of one of the situations:

- First part of the situation: https://youtu.be/oQs2QQueKOk
- Second part of the situation: https://youtu.be/Cnj380M9SHE
- Third part of the situation: https://youtu.be/FtNACAAnEOw

The structure of the situations is as follows:

1- Office: The participant starts de experience in an office where is alone:

- The participant is asked a question that is more or less emotionally charged according to the situation. Example: “What problems do you find in achieving your goals and how do you think you could solve them? vs “how can you define your relationship with others? If you could change a few things about the relationships you have, how would you like it to be?”

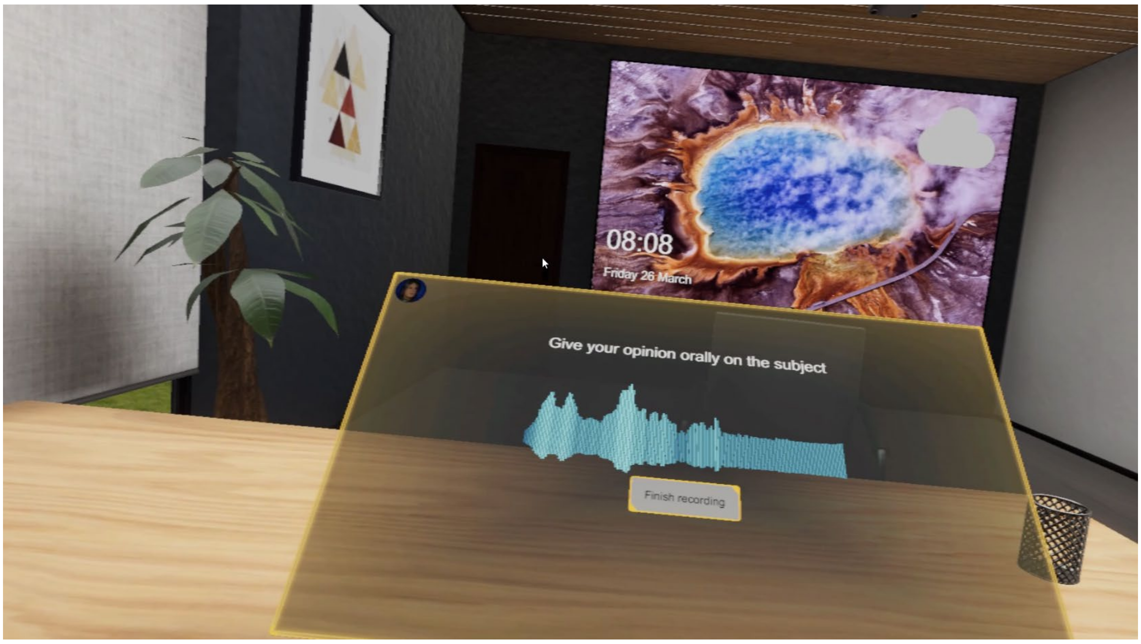

- Two tasks of interaction with the environment are presented: Receive messages by group chat and e-mail messages. The content of messages and emails differs in situations.
  - Receive messages by group chat: The participant has to decide whether or not to answer the messages. This is a measure of how engaged the participant is in personal aspects while is immersed in the work environment. Examples of chat messages are: "Trivial topics such as internet jokes and images with humorous content" vs "raise logical problems, raise personal problems and praise/criticism".

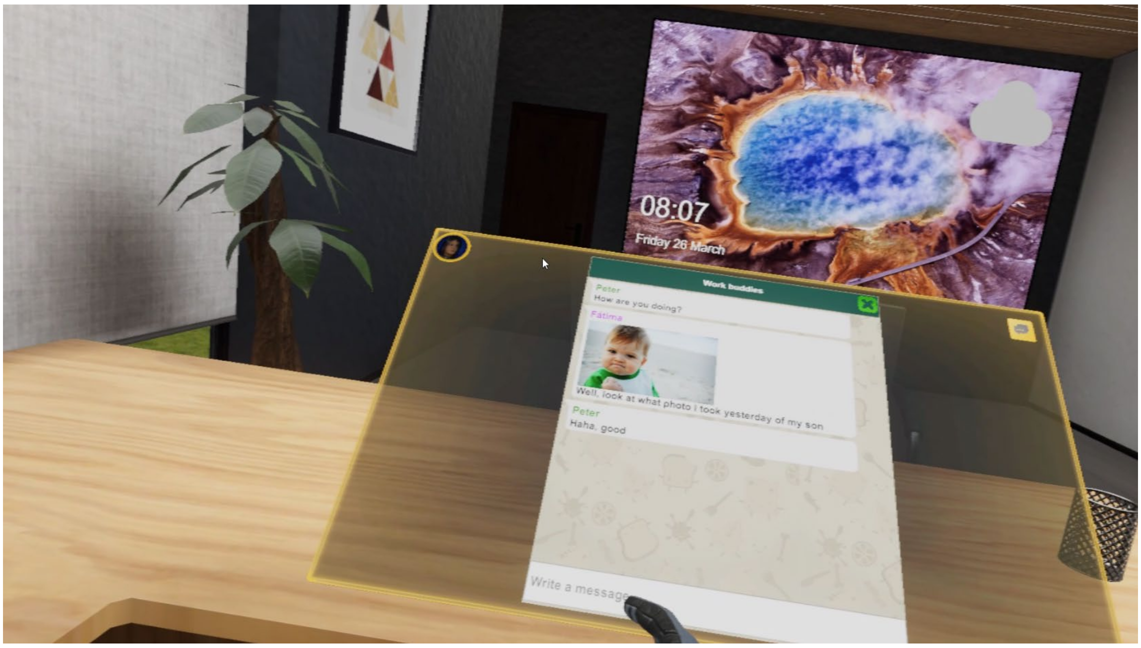

- Receive email messages: The content of these emails is associated with tasks that attempt to capture the empathic skills of the participant and, therefore, the ability to recognize emotions. Examples of these tasks: Photos of faces with different facial expressions (It is about identifying the emotions expressed in the faces) Video without sound of people talking (It is about paying attention to the non-verbal behaviour of the characters to try to identify their emotions) and audio without video (It is about identifying emotions only through the voice of the characters).

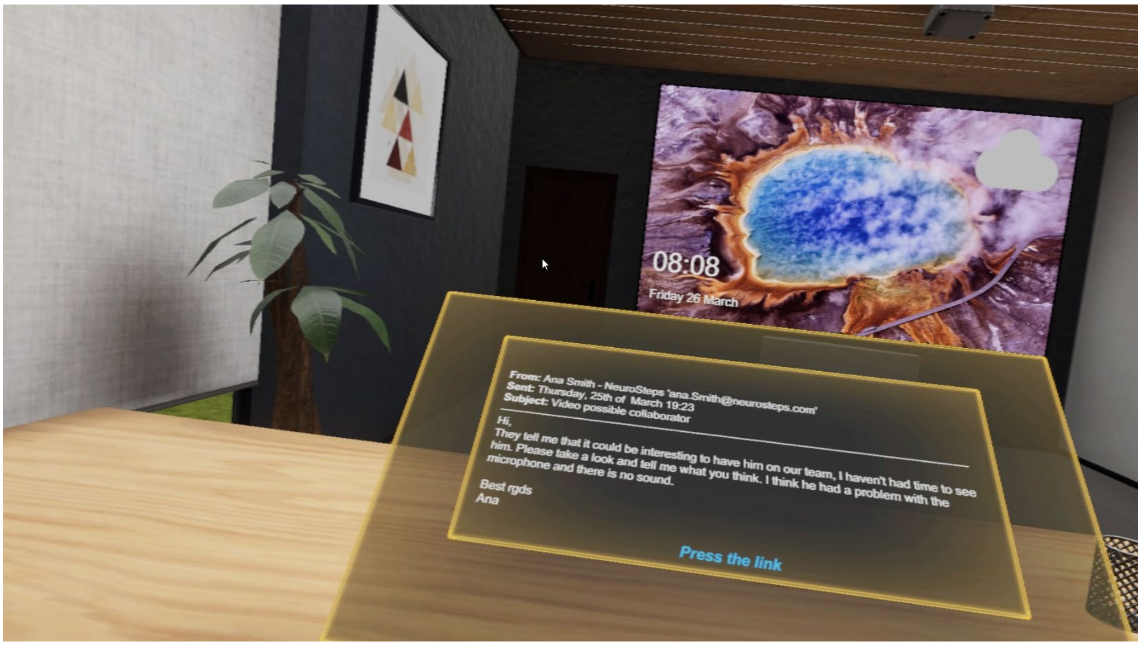

2- Meeting room: The participant is in a meeting room with more characters

- The participant is asked to select the place that want to occupy at the meeting table. There are three options: chair the table, next to an avatar, or stand by the door.

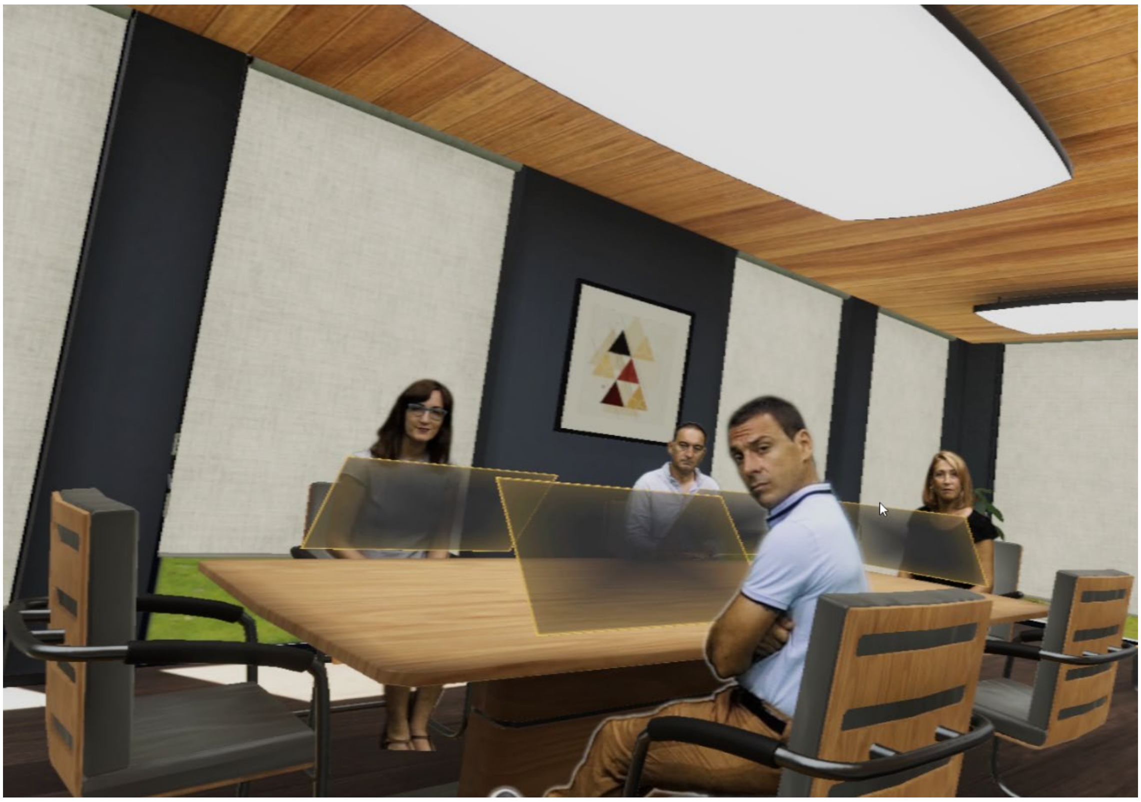

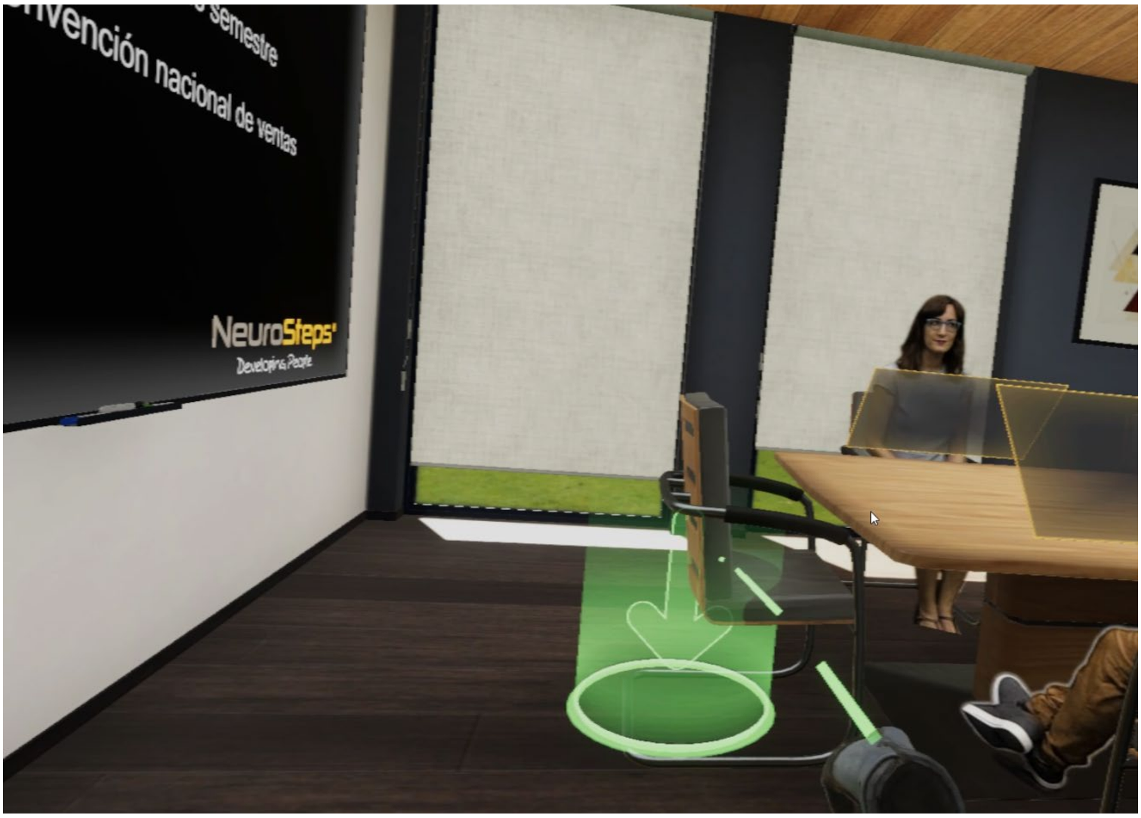

- Two or three meeting topics are presented. The topics have different emotional charge. Example “Organize meeting calendar” vs “Heated discussion where the center of the problem is the participant”. All the avatars interact and the user must present his own opinion by voice and then must select the option that best suits his/her response, and therefore, the one that best suits his/her behavioural style and personality)

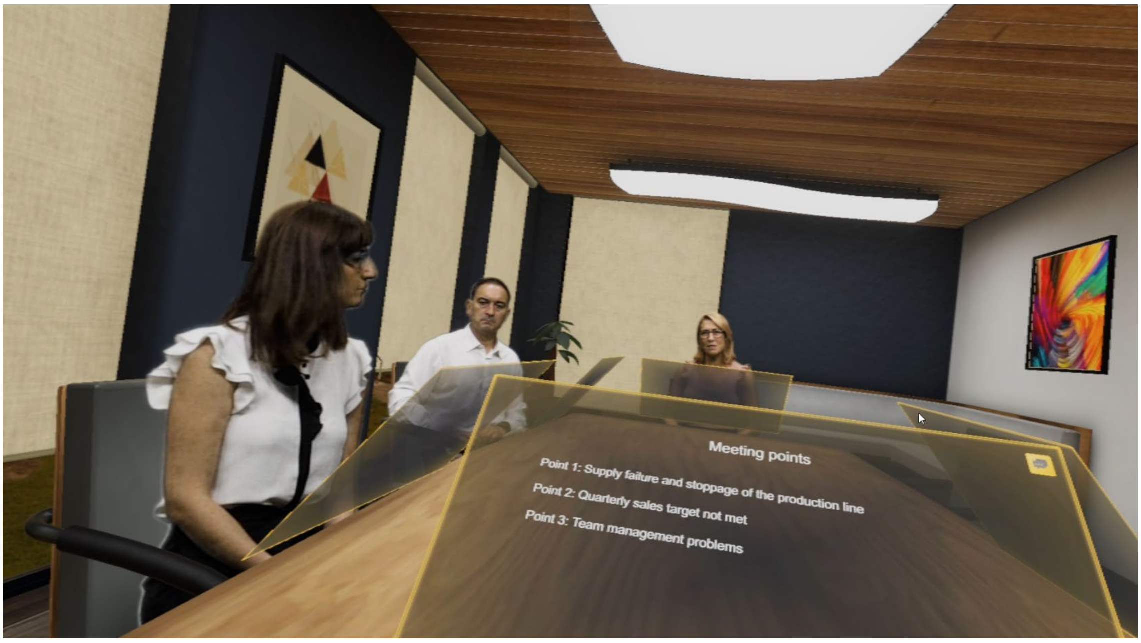
Meeting points
Point 1: Supply failure and stoppage of the production line
Point 2: Quarterly sales target not met
Point 3: Team management problems

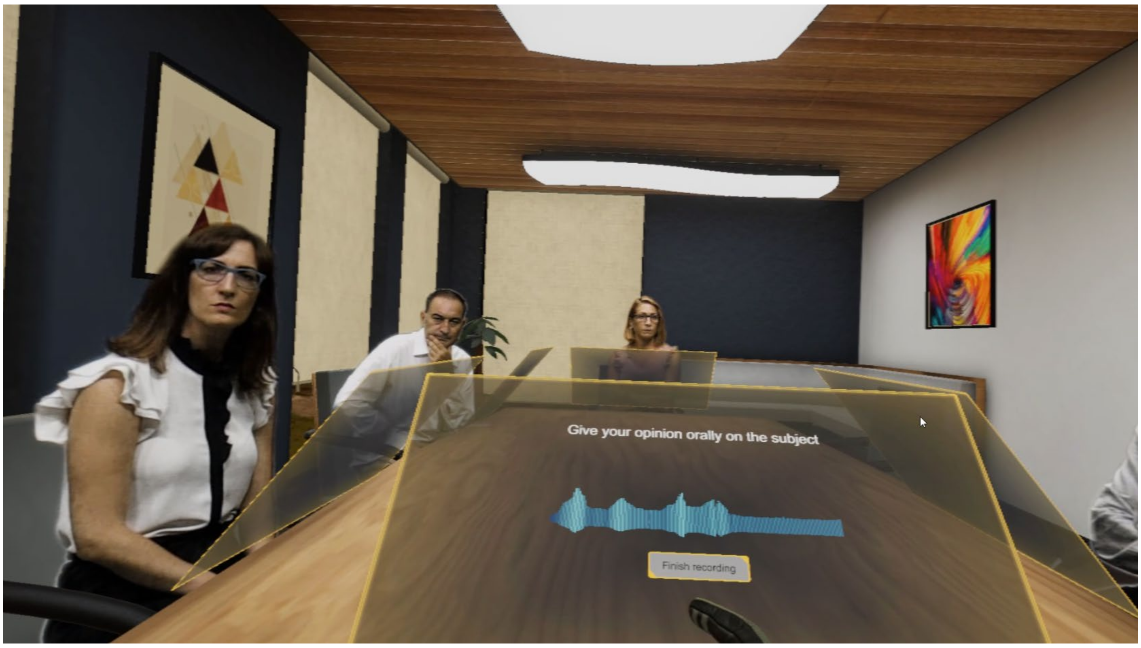
Give your opinion orally on the subject
Finish recording

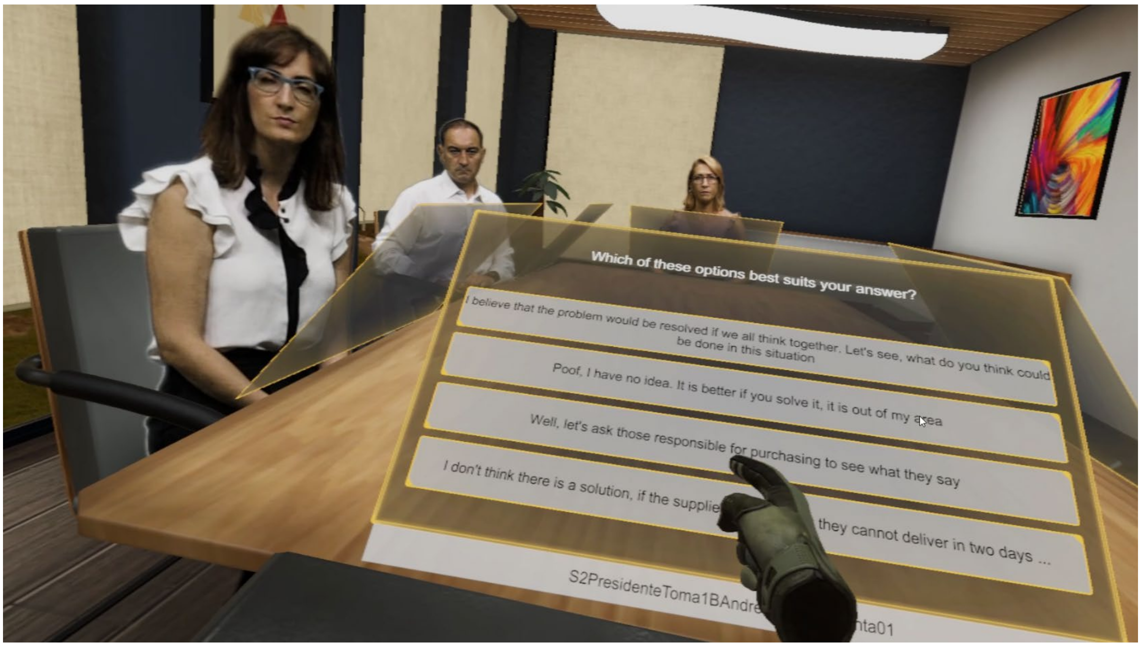

- Throughout the development of the different meeting points, a series of intermediate tasks are presented: Group chat messages with different topics that the user can answer or ignore and a series of situations that participants have to resolve (decision-making tasks):
    - Receive messages by group chat: The participant has to decide whether to answer the messages or not. This task seeks to obtain the degree of commitment and involvement of the participant in the meeting.

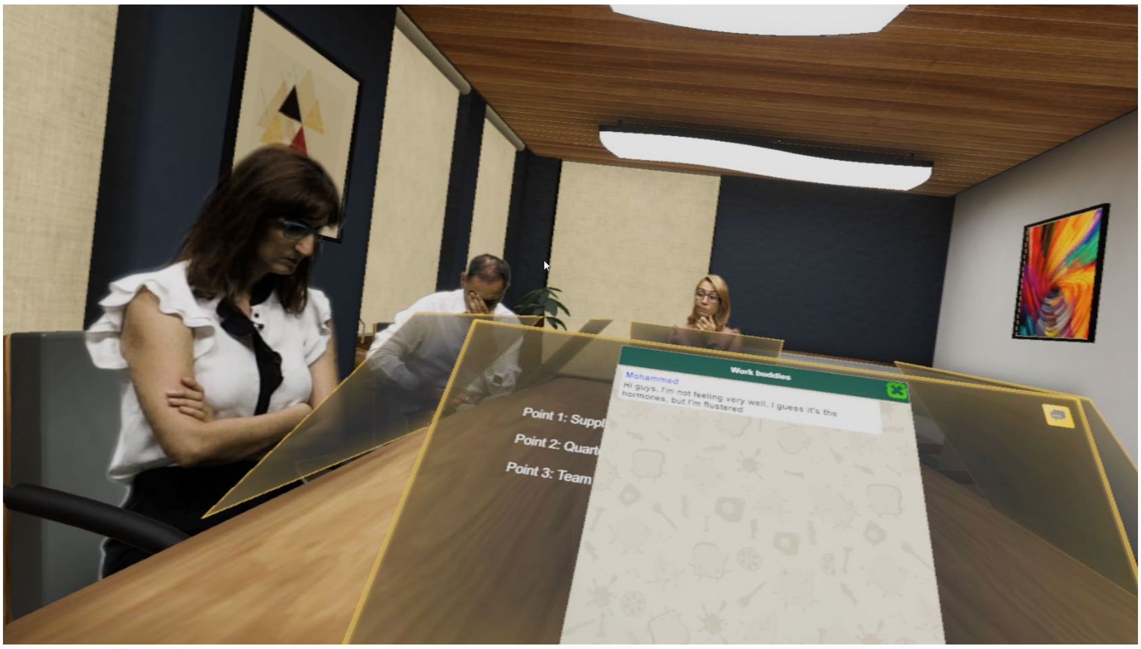

- Receive messages with a series of situations that the participants must solve. The objective of these tasks is to capture the decision-making style of the participants. An example of the situations is: “You are playing a team game and you are the team leader. For the next test, you have to build an object and decide the roles. The final decision is yours."

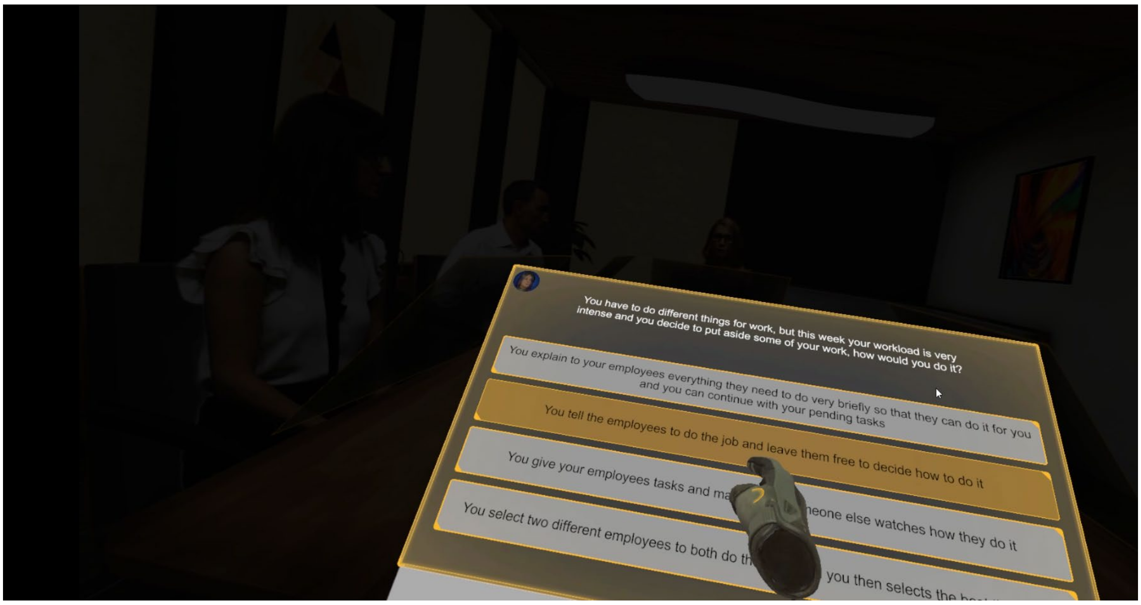

- Depending on the option chosen, a mini-game will appear after the decision.

3- Office: Once the meeting is finished, the participant returns to the office

- The participants are asked to rate their behaviour and involvement in the group chat.

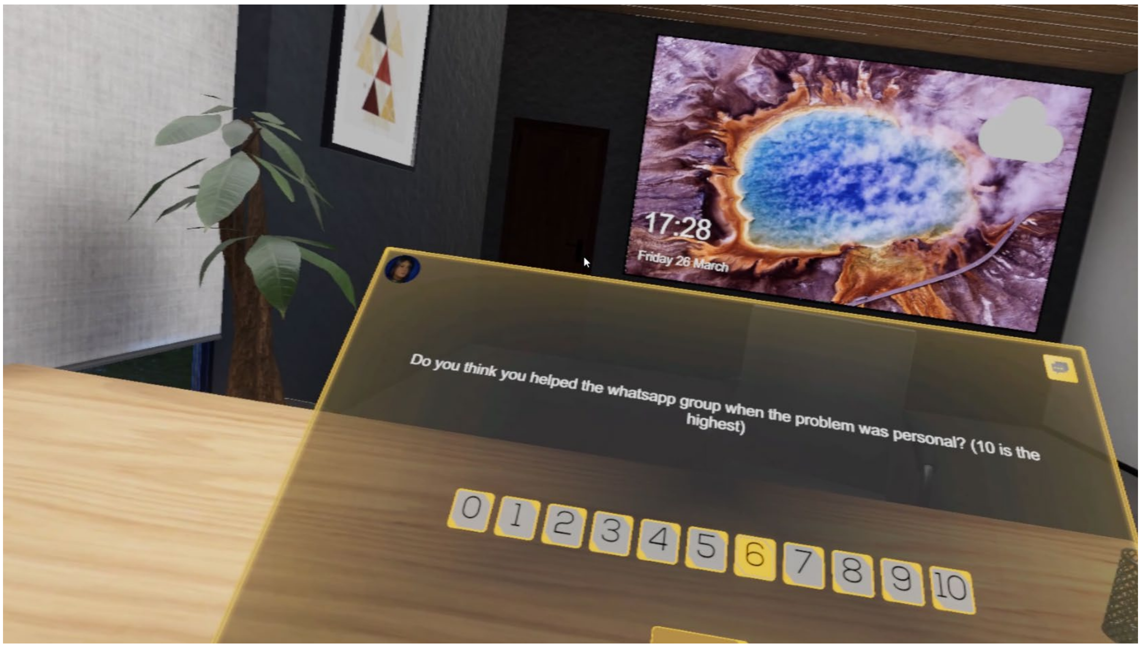

- Once their behaviour is rated, a series of mini-games appear in an email. The participant is instructed to select three of these tasks. Once selected, a tutorial appears explaining each of the tasks to be performed. After this, the participant is encouraged to perform each task. Once completed, the user is asked how they rate each of the tasks (positive or negative). In this way, it can be observed if the user perceives the tasks as stress or as pleasure (work or challenge).

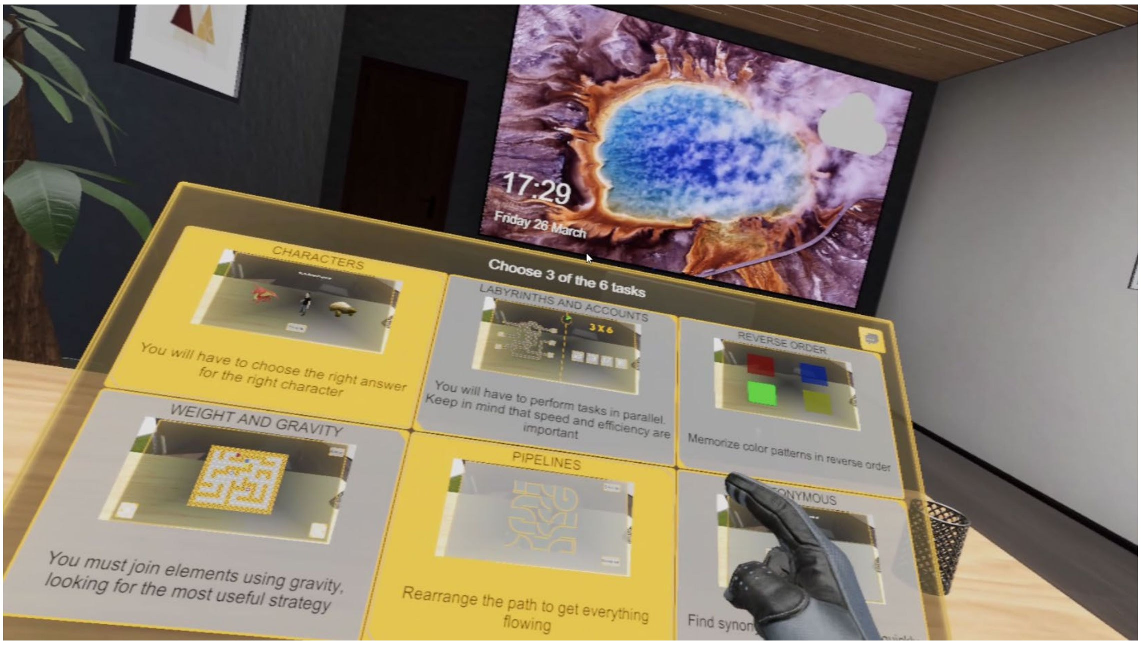

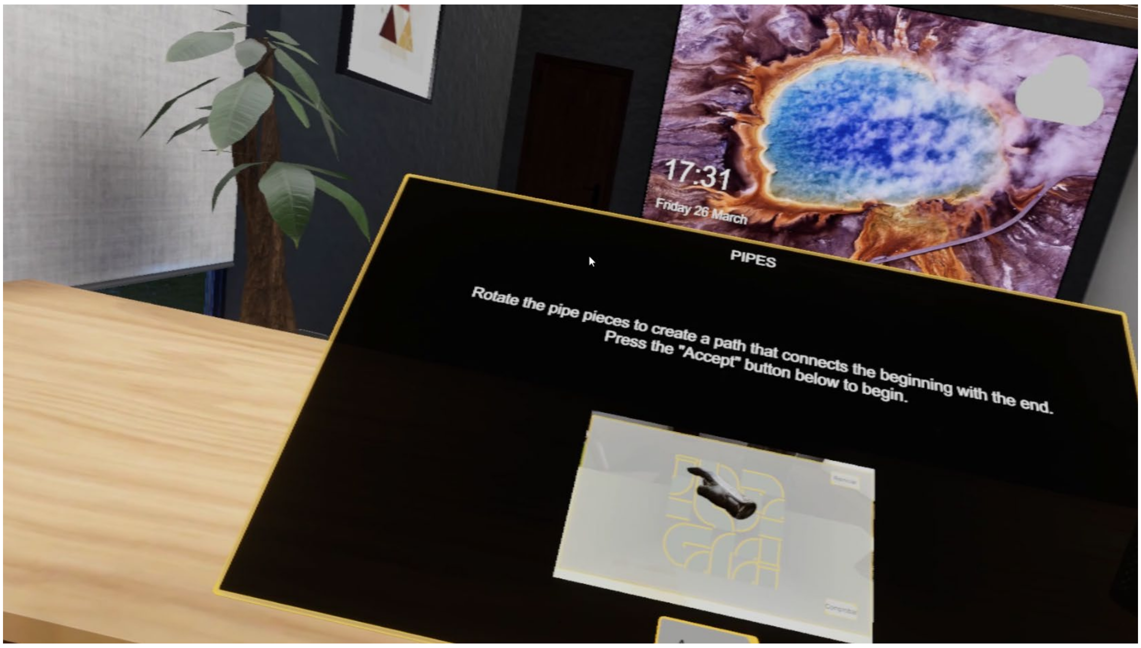

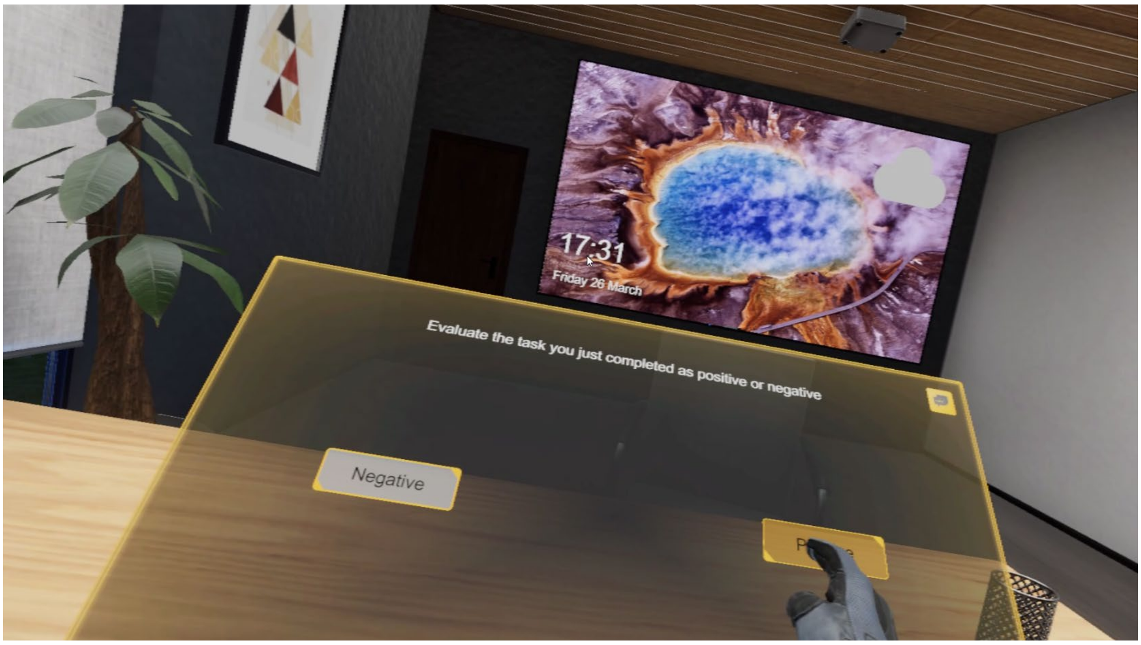

Next, each of the tasks that have been evaluated for the analysis and study of the different leadership styles are presented according to the previous structure. All this information was recorded simultaneously with the eye-tracking signal throughout the virtual reality experience. For

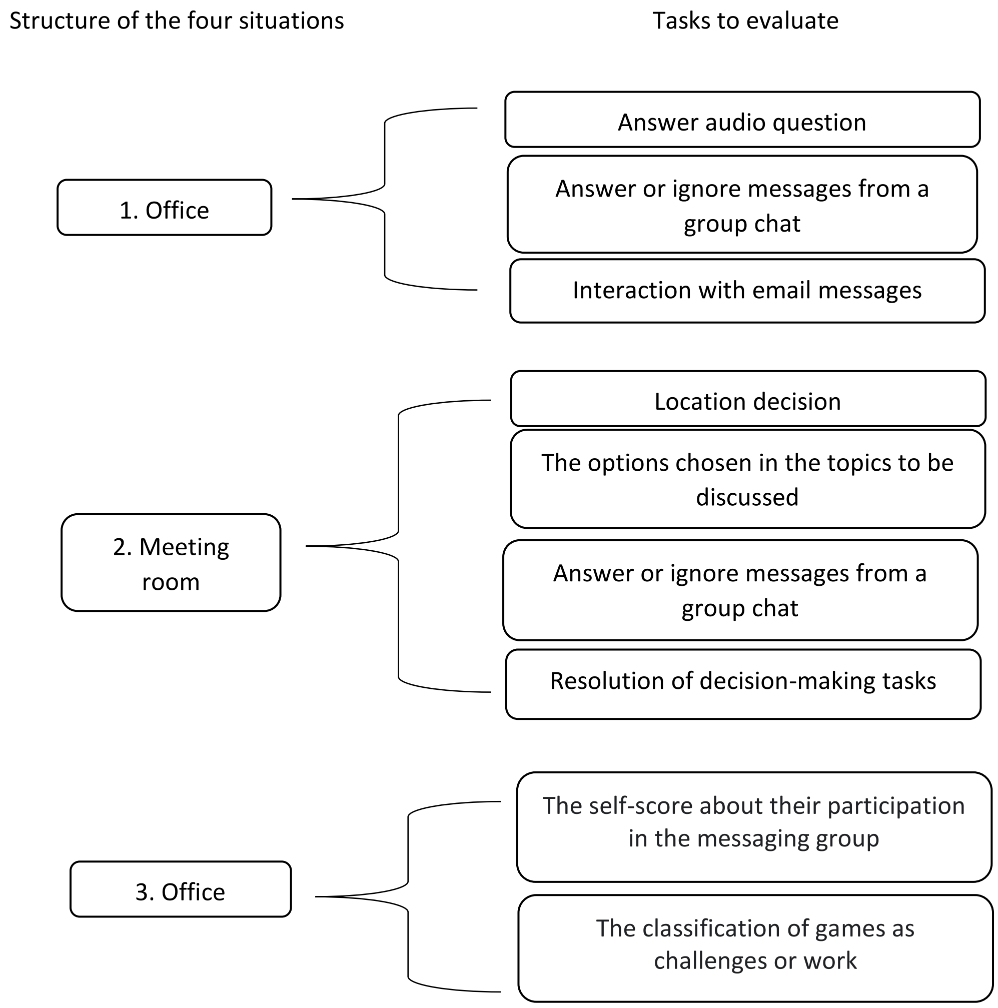
Structure of the four situations
Tasks to evaluate
1. Office
Answer audio question
Answer or ignore messages from a group chat
Interaction with email messages
2. Meeting room
Location decision
The options chosen in the topics to be discussed
Answer or ignore messages from a group chat
Resolution of decision-making tasks
3. Office
The self-score about their participation in the messaging group
The classification of games as challenges or work
